# Uptake of pre-cervical cancer screening and associated factors among reproductive age women in Debre Markos town, Northwest Ethiopia, 2017

**DOI:** 10.1186/s12889-019-7398-5

**Published:** 2019-08-14

**Authors:** Simachew Animen Bante, Simegnew Asmer Getie, Almaz Aklilu Getu, Kebadnew Mulatu, Selamawit Lake Fenta

**Affiliations:** 1 Department of Midwifery, College of Medicine and Health Sciences, Bahir Dar University, Bahir Dar, Ethiopia; 2 Department of Public Health, College of Medicine and Health Sciences, Bahir Dar University, Bahir Dar, Ethiopia

**Keywords:** Pre-cervical cancer, Reproductive health, Pre-cervical cancer screening, Health education

## Abstract

**Background:**

Cervical cancer remains a major cause of morbidity and mortality among women, particularly in low-resource countries like Ethiopia. Early screening for pre-cervical cancer is a key intervention in reduction of maternal deaths. We assessed uptake of pre-cervical cancer screening and its associated factors among women of reproductive age in Debre Markos town in northwest Ethiopia.

**Methods:**

A community-based, cross-sectional study was conducted among 517 women of reproductive age. A multistage sampling technique was used to select study participants. *Kebeles* (sub-districts) were selected by a simple random sampling technique.Data was entered using Epi Info and analyzed by SPSS. Variables in binary logistic regression with a *P* value < 0.2 were fitted to multivariable logistic regression. Significant variables were declared at 95% CI and an AOR of P value < 0.05.

**Results:**

A total of 517 women were interviewed with100% response rate. Results revealed only 108 women (20.9%) [95% CI =17.6–24.6] had been screened for pre-cervical cancer. Participants ages 35–49 [AOR = 3.21, 95% CI: 1.40, 7.39] informed by health professionals about cervical cancer [AOR = 6.65, 95% CI: 3.64, 12.15], positive attitude to screening [AOR = 3.38, 95% CI: 1.92, 7.61], visited health institution once or more in a year [AOR = 6.72, 95% CI: 2.40, 18.79], visited health institution once or more in two years [AOR = 3.76, 95% CI = 1.39, 10.19], history of sexually transmitted infections [AOR = 2.37, 95% CI: 1.11, 5.07] and family history of cervical cancer [AOR = 4.95, 95%CI: 1.62,15.15] were significantly associated with pre-cervical cancer screening.

**Conclusion:**

Uptake of pre-cervical cancer screening was found to be low among women of reproductive age. Age, attitude, informed by health provider, visiting health institution, history of sexually transmitted infections and family history of cervical cancer were found to be significantly associated with higher uptake of screening. To scale up currently limited uptake of pre-cervical cancer screening, community health education should be undertaken, leading to attitude change for young women.

## Background

Cervical cancer is the second commonly diagnosed cancer and the leading cause of cancer death in African women [1]. The prevalence of cervical cancer is high in low- and middle-income countries due to lack of awareness for cervical cancer screening, higher rate of Human Papilloma Virus (HPV) infection and inaccessibility of screening [2].

Every year more than 270,000 women die worldwide from cervical cancer [3]. Cervical cancer is usually detected in advanced stages in developing countries due to lack of effective preventive mechanisms. An estimated 80%of all patients with cancer in developing countries are presented with advanced stages at their first visit [1].Poor access to screening and treatment services attributed to more than 85% of women’s death in low- and middle-income countries [4]. Death rates vary from country to country. This is due to lack of screening services for the prevention and early detection of the disease [1].

Even with the presence of these major problems, only few women receive pre-cervical cancer screening services in Ethiopia. Uptake remains low due to screening methods -only visual inspection with acetic acid (VIA) and because the test is not patient-friendly in terms of privacy [5].

For the past many years, high-income countries have seen a dramatic drop in cervical cancer incidence and mortality. However, similar success has not yet been achieved in low- and middle-income countries like Ethiopia. This is largely due to little information about pre-cervical cancer screening. In many countries, offering VIA to all young adolescent girls and preventive treatment programs has been radically increased. But much remains to be done, especially in the lower-income countries like Ethiopia where the burden of cervical cancer is highest [4].

Maternal morbidity and mortality related to cervical cancer is the second most occurring and the leading killer of women among all cancer types in Ethiopia. Studies have shown that uptake of pre-cervical cancer screening is very low, for example, in the towns of Arbaminch (5.9%) and Dessie (11%) [6, 7] compared to the National cervical cancer prevention strategic plans (80%) set by Federal Ministry of Health of Ethiopia [8]. Additionally, the factors associated with low coverage have not been well investigated. Therefore, engagement in cervical cancer prevention and screening could result in one of the most significant successes to decrease women mortality and morbidity related to cervical cancer in Debre Markos town as well as in the country [5].

Most pre-cervical cancer screening studies in Ethiopia have been conducted on knowledge and attitude. Only a few studies were conducted on pre-cervical cancer screening practice and associated factors. Hence, there is limited evidence on the uptake of pre-cervical cancer screening(PCCS) and associated factors in most regions of Ethiopia [9]. This community-based study will play vital role in generating data that is important to fill gaps for program and policy makers. It can also be a source for advancing further study in the field of cervical cancer prevention.

## Methods

### Study setting, period and participants

A community based, cross-sectional study was conducted from March 01, 2017 to April 01, 2017 in Debre Markos town, capital of East Gojjam zone, northwestern Ethiopia. The town is located at 300 km far from Addis Ababa, capital of Ethiopia and 265 km from Bair Dar, capital of Amhara regional state. According to the 2015 Census, the town has a total population of 113,101, of which, 60,425 were women. There are seven *kebeles*, one referral hospital, three health centers and five nongovernmental clinics which are giving different reproductive health services in the town. It is only the government hospital that gives pre-cervical cancer screening service. All women in reproductive age group who are living in Debre Markos town were source populations. Therefore, all women in reproductive age group who were available during data collection period in selected *kebeles* were study populations. In this study, women who had been living in Debre Markos town for at least six months and age group from 15 to 49 years old were included. Women who were seriously ill and women who had total abdominal or vaginal hysterectomy were excluded.

### Sample size and sampling procedure

The sample size was calculated by using the single population proportion formula with the assumption of 95% CI, and 11% proportion of women who had undergone pre- cervical cancer screening in Dessie town [10]. 10% non-response rate and 4% margin of error was used to obtain sample size of 235. Since the sampling technique was multistage, design effect of 2 was considered as well as with adjustment of 10% non- response rate, the final sample size was 517. Four *Kebeles* were selected by simple random sampling technique. The total sample size was proportionally allocated for each *Kebele*. Study subjects were selected by systematic sampling technique. Sampling fraction was determined by dividing the total households in each *kebele* for the sample size which was proportionally allocated in each *kebele*. The first household was selected by lottery method and then every 27th interval in each *kebele* was included in the study. Women within reproductive age group were interviewed from the selected households and if there were more than one woman in the household, lottery method was used to select one.

### Data collection tools and techniques

The questionnaires were adapted from related study [11]. It was first prepared in English and then translated in Amharic (local language) and back translated into English by language experts to ensure uniformity. Interviewer administered pre-tested questionnaire was used for data collection. The data were collected by four diploma midwives and supervised by two BSc midwives. Data collectors and supervisors were trained for two days prior to data collection. Pre-test was done on 26 individuals other than selected *kebeles*. Personal identifiers were not included in the questionnaires to ensure participants confidentialities. The questionnaires were checked on daily bases by supervisors and principal investigator.

### Variables and measurements

Independent variables in this study were classified into 5 sections: these were socio- demographic characteristics, reproductive characteristics, knowledge related variables, attitude related variables to pre-cervical cancer screening, visiting health facility and health providers related variables were used to assess the uptake of pre-cervical cancer screening in this study. The dependent variable in this study was ever being screened for pre-cervical cancer screening.

Twenty three questions were asked on knowledge of pre-cervical cancer screening: source of information about pre-cervical cancer screening, symptoms of cervical cancer, screening practice and causes of cervical cancer. The response to each of the questions was “yes” or “no”. The responses were scored and summed. Each response was given a score of one for correct answers and zero for incorrect once. The cut-offs were defined knowledgeable and not knowledgeable using median scores.

The attitude of the participants about pre-cervical cancer screening were early prevention, the burden of the cervical cancer, severity, susceptibility, barriers for screening, results of pre-cervical cancer screening and treatment were measured using Likert scale which ranges from score 4 (strongly agree) to score 1 (strongly disagree). The responses were summed up and a total score was obtained for each respondent. The median score was calculated and those who scored above median score of attitude assessing questions were categorized as having positive attitude about pre-cervical cancer screening and those who scored the median value or less than the median of attitude assessing questions were categorized as having negative attitude about pre-cervical cancer screening.

#### Reproductive age women

Women aged from 15 up to 49 years old [12].

#### Screening uptake

The proportion of women who have ever been screened for pre-cervical cancer at least once in life time [13].

#### Knowledgeable

Study participants who scored above the median score of 23 knowledge assessing questions [14].

#### Not knowledgeable

Study participants who scored the median value or less than the median score of 23 knowledge assessing questions [14].

#### Positive attitude

Study participants who scored above median score of 7 attitude assessing questions [14].

#### Negative attitude

Study participants who scored the median value or less than the median of 7 attitude assessing questions [14].

### Data processing and analysis

Data entry and cleaning was done with Epi Info 7. Data were exported to SPSS version 21 for analysis. Descriptive statistics were analyzed and presented using tables and figures. The outcome variable is binary in its nature and it is labeled as “1” when the participants were screened for PCCS, otherwise “0”. Frequencies and proportions were computed for description in relation to socio-demographic and other variables. Strength of statistical associations was determined using adjusted odds ratio with 95% confidence interval. Relationships between each independent variable and outcome variable were investigated using binary logistic regression model. Those variables with P- value less than 0.2 at the bivariable level were included in a multivariable logistic regression model for controlling potential confounding effect. In the multivariable analysis, variables with p- value < 0.05 were considered as associated factors.

## Result

### Socio-demographic characteristics

A total of 517 reproductive age group women interviewed making 100% response rate. The mean age of the participants was 30.1 years (SD ±7.9). Two hundred twenty eight (44.1%) of them were in the age group of 25–34 years (Table 1).

**Table 1:** Distribution of study participants by their socio-demographic characteristics in Debre Markos town, northwestern Ethiopia, March– April2017 (*n* = 517)

Age	Frequency	Percent
15–24	134	25.9
25–34	228	44.1
35–49	155	30
Ethnicity		
Amhara	495	95.7
Oromo	11	2.1
Tigre	9	1.7
Others^a^	2	.4
Religion		
Orthodox	441	85.3
Muslim	44	8.5
Protestant	23	4.4
Catholic	9	1.7
Marital status		
Married	321	62.1
Single	116	22.4
Windowed	29	5.6
Divorced	51	9.9
Education		
Unable to read and write	96	18.6
Able to read and write	74	14.3
Primary (1–8)	82	15.9
Secondary (9–12)	118	22.8
Collage and above	147	28.4
Occupation		
Merchant	115	22.2
Housewife	146	28.2
Governmental employee	140	27.1
Farming	39	7.5
Student	50	9.7
Others^b^	27	5.2
Household income		
< 867 ETB	172	33.3
867–2500 ETB	180	34.8
> 2500 ETB	165	31.9

^a^Gumuz (*n* = 1) and Agew (*n* = 1)

^b^Daily laborer (*n* = 22), Waiter, (*n* = 2) and Commercial sex worker (*n* = 3)

### Reproductive characteristics

More than half of the participants 297 (57.4%) had single sexual partner. 443 (85.7%) of participants and most 511 (98.8%) had no history of STI and smoking respectively (Table 2).

**Table 2:** Reproductive Characteristics of the study participants in Debre Markos town, Northwest Ethiopia, March– April 2017 (*n* = 517)

Variable		Frequency	Percent
Age at first intercourse	< 18	236	45.6
	> = 18	281	54.4
Have you given birth?	Yes	336	65
	No	181	35
Number of children	< 2	317	94.3
	> = 2	19	5.7
HIV test	Yes	389	75.2
	No	128	24.8
HIV test result	Negative	335	86.1
	Positive	54	13.9
History of STI	Yes	74	14.3
	No	443	85.7
History of cervical cancer in family	Yes	36	7
	No	481	93
Lifetime Number of sexual partners	Single partner	297	57.4
	Multiple partner	220	42.6
History of contraceptive use	Yes	277	53.6
	No	240	46.4
History of smoking	Yes	6	1.2
	No	511	98.8

### Knowledge and attitude of participants about pre-cervical cancer screening

**Fig. 1:**
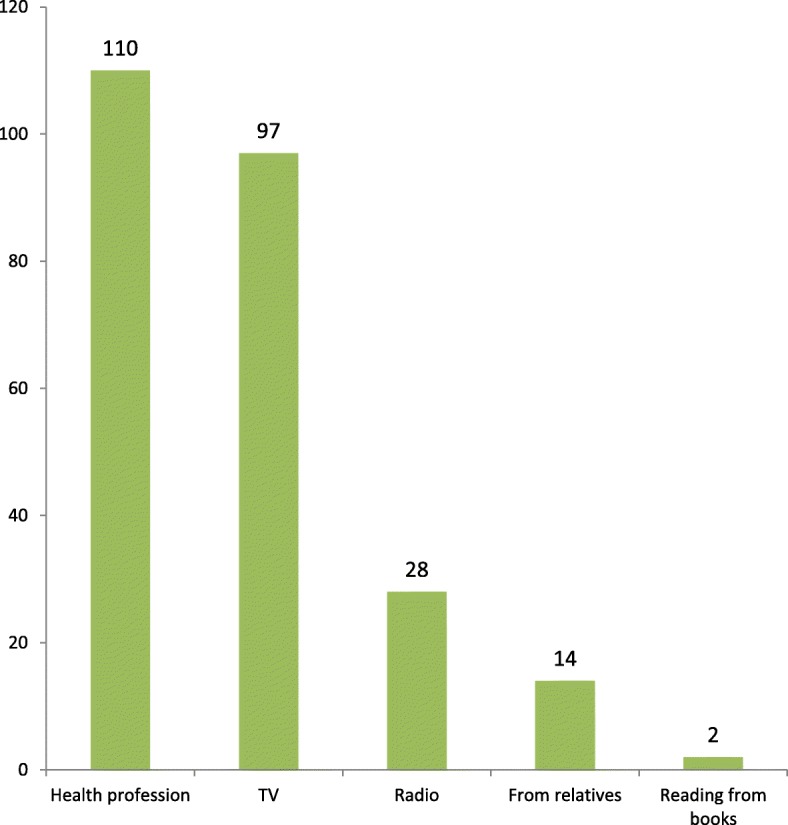
Source of information for study participants regarding to pre-cervical cancer screening in Debre Markos town, northwestern Ethiopia, March– April2017 (*n* = 251)

In this study, 240 (46.4%) of participants were knowledgeable about pre-cervical cancer screening. More than half 266 (51.5%) of participants did not hear about pre-cervical cancer screening. The dominant source of information was health professionals 110 (43.8%) followed by television 97 (38.6%) (Fig. 1).

Two hundred twenty-nine (44.3%) participants had positive attitude about pre-cervical cancer screening.

### Uptake of pre-cervical cancer screening

**Fig. 2:**
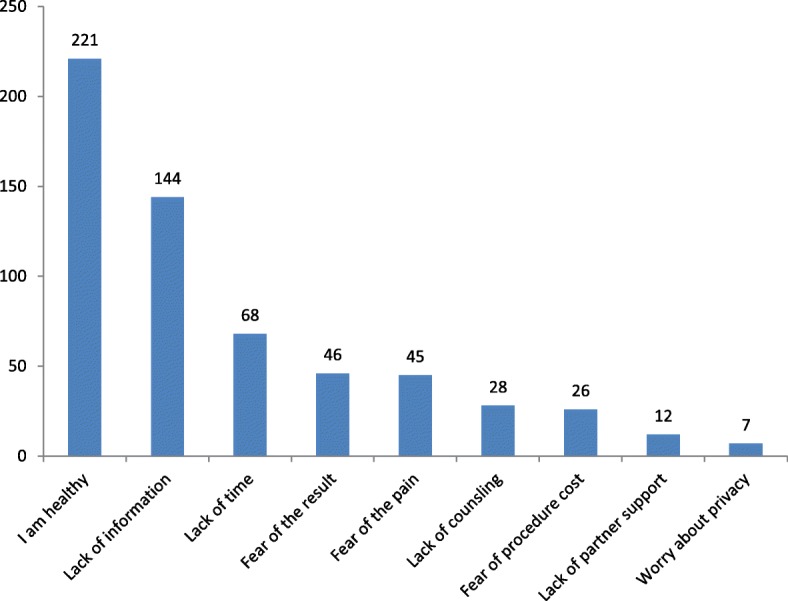
Main reasons not to be screened for pre-cervical cancer among who had never screened in Debre Markos town, northwestern Ethiopia, March – April2017 (*n* = 409)

In this study, uptake of pre-cervical cancer screening was 108 (20.9%). The reasons mentioned by participants for not being screened were being healthy (42.7%) followed by lack of information about PCCS (27.9%), (Fig. 2).

### Factors associated with uptake of pre-cervical cancer screening

In bivariable analysis; age, income, informed about cervical cancer by health professionals, knowledge of women on cervical cancer, given birth, number of partner, knew someone diagnosed with cervical cancer, attitude of women on cervical cancer, history of STI, family history of cervical cancer, and visiting health institution were statistically significant with the uptake of pre-cervical cancer screening.

Multivariable logistic regression showed age, informed about cervical cancer by health professionals, attitude of women on cervical cancer, family history of cervical cancer, history of STI and frequency of visiting health institutions were associated with uptake of pre-cervical cancer screening (Table 3).

**Table 3:** Bivariable and Multivariable analysis of factors associated with uptake of pre-cervical cancer screening (PCCS) in Debre Markos town, northwest Ethiopia, March– April2017 (n = 517)

Variable	PCCS		COR (95%CI)	AOR (95%CI)	*P* value
	Yes	No			
Age					
15–24	15	119	1	1	
25–34	36	192	1.49 (0.78, 2.83)	1.50 (0.67, 3.33)	
35–49	57	98	4.61 (2.46, 8.65)	3.21 (1.40,7.39)	.006
Income, ETB (Ethiopian Birr)					
< 867 ETB	24	148	1	1	
867–2500 ETB	31	149	1.28 (0.72, 2.29)	1.28 (0.59, 2.78)	.541
> 2500 ETB	53	112	2.92 (1.70, 5.03)	1.93 (0.89, 4.21)	.098
History of STI					
Yes	44	30	8.69 (5.09, 14.82)	2.37 (1.11, 5.07)	.026
No	64	379	1		
Have you given birth					
Yes	89	247	3.07 (1.80,5.24)	1.20 (0.53,2.64)	.658
No	19	162	1		
Family history of cervical cancer					
Yes	27	9	14.8 (6.72, 32.69)	4.95 (1.62,15.15)	.005
No	81	400	1		
Number of partners					
Single	51	246	1		
Multiple	57	163	1.69 (1.10, 2.59)	1.04 (0.55,1.95)	.908
Knowledge of pre-cervical cancer					
Knowledgeable	87	153	6.93 (4.13, 11.62)	0.59 (0.27, 1.34)	.210
Not knowledgeable	21	256	1		
Attitude of women on pre-cervical cancer					
Positive attitude	93	136	12.45 (6.95, 22.29)	3.38 (1.92,7.61)	.000
Negative attitude	15	273	1		
Ever visited health institution					
Once or more in one year	55	69	19.49 (9.16,41.45)	6.72 (2.40,18.79)	.000
Once or more in two years	44	120	8.96 (4.23,18.99)	3.76 (1.39,10.19)	.009
Never visited	9	220	1		
Informed about PCCS by health provider					
Yes	85	90	13 (7.81, 21.96)	6.65 (3.64,12.15)	.000
No	23	319	1		
Knew someone with cervical cancer					
Yes	59	52	8.27 (5.13, 13.33)	1.15 (0.56,2.36)	.700
No	49	357	1		

This study found that those women who were in the age group of 35–49 years were 3.21 times more likely to take pre-cervical cancer screening than those whose ages were between 15 and 24 years [AOR = 3.21, 95% CI: 1.40, 7.39].

Those women who were informed by health professionals about cervical cancer were 6.65 times higher to take pre-cervical cancer screening as compared to those who were not informed [AOR = 6.65, 95% CI: 3.64, 12.15].

Women who had positive attitude about cervical cancer were 3.38 times more likely to take pre-cervical cancer screening than their counter parts [AOR = 3.38, 95% CI: 1.92, 7.61].

Participants who had visited health institutions once or more in a year were 6.72 times higher and those who had visited health institution once or more in two years were 3.76 times higher to be screened as compared to those who never visited [AOR = 6.72, 95% CI: 2.40, 18.79 and [AOR = 3.76, 95% CI: 1.39, 10.19] respectively.

Participants who had history of STI were 2.37 times more likely to take pre-cervical cancer screening than those who had no history of STI [AOR = 2.37, 95% CI: 1.11, 5.07].

Participants who had family history of cervical cancer were 4.95 times higher to use pre-cervical cancer screening when compared to those who had not [AOR = 4.95, 95% CI: 1.62,15.15].

## Discussion

The uptake of pre-cervical cancer screening among women of reproductive age was 20.9% (95% CI =17.6–24.6%), which is lower than the Federal Ministry of Health National cervical cancer prevention strategic plans (80%) [8].

The result is in line with studies done in Kenya (25%), Tanzania (21%) and in Ethiopia (19.8%) [11, 15, 16]. However, this finding was lower than the study conducted in Thailand (65.9%), Jamaica (66%) and in Kenya (39%) [17–19]. The reason may be due to accessibility of the service, having many methods of pre-cervical cancer screening and availability of more trained health providers. It may also be due to differences in socio-demographic characteristics such as the level of education of the study participants in the countries. Age of the study participants might also be another possible reason. The method of the study may also contribute to the difference. In a facility-based study as participants might get information from health providers, it increases the uptake of pre-cervical cancer screening. This finding was higher than the study conducted in Nigeria (8%) [20], Uganda (4.8%) [21], Dessie (11%) [10] and Arbaminch, Ethiopia (5.9%) [6]. This difference might be brought by age and other socio-demographic characteristics difference of study participants. The difference could also partially explain by the time gap between the studies as well as the study area might be different among the studies.

This study showed that maternal age was one of the significant predictors of pre-cervical cancer screening uptake. Women in the age group 35–49 were 3.21 times more likely to be screened as compared to mothers in the age group 15–24 (AOR = 3.21, 95% CI = 1.40–7.39). This finding is supported with previous studies conducted in Greece, Jamaica, Mekelle, Arbaminch and Dessie [6]. The possible explanation could be that cervical cancer symptoms usually develop in women above 30 year sold. Additional explanations may be that Ethiopian health policy encourages screening of more than 30 years old women. It may also be due to the fact that as age increases they may be multipara so they might visit health institution for antenatal care, post-natal care and for delivery, while at the same time receiving information about cervical cancer.

History of sexually transmitted infections was a significant factor for pre-cervical cancer screening uptake. Women who had a history of sexually transmitted disease were 2.37 times more likely to be screened as compared to those participants who had no history of sexually transmitted disease (AOR = 2.37, 95% CI =1.11–5.07). This study is in line with studies done in Kenya, Kasarani and Mekelle [11, 22]. When women are treated for STIs at institutions, they could be told about the relationship of cervical cancer and STIs by the health provider so that they could get screened. These participants might also be near for reproductive health services so that they might further check other reproductive services like cervical cancer screening.

Family history of cervical cancer was other significant factor for uptake of pre-cervical cancer screening. Those participants who had history of cervical cancer in their family were 4.95 times more likely to be screened as compared to their counterparts (AOR = 4.95, 95% CI = 1.62–15.15). This is the first report and there are no other studies that support those participants who had family history of cervical cancer were more likely to screen pre-cervical cancer screening. The possible reason might be that women who had family history of cervical cancer would think that cervical cancer will be inherited genetically so that they would screen by fear of this.

Attitude of participants was significant predictor of pre-cervical cancer screening. Participants who had positive attitude were 3.38 more likely to be screened than those participants who had negative attitude (AOR = 3.38, 95% CI = 1.92–7.61). This finding is in line with studies conducted in Ilorin, Nigeria and Dessie, Ethiopia [20, 23]. Anticipated reasons for this might be those participants who had positive attitude about cervical cancer could believe pre-cervical cancer screening will prevent from developing cervical cancer.

Visiting health institutions was also significant factor for pre-cervical cancer screening. Those participants who were visiting a health institution for any reason once or more in a year were 6.72 times higher (AOR = 6.72, 95% CI = 2.40, 18.79) and once or more in two year were 3.76 times higher (AOR = 3.76, 95% CI = 1.39, 10.19) to be screened as compared to those participants who never visited health institution. This finding is in agreement with previous studies done in Jamaica and Kenya [24, 25]. The explanation might be that women who had visited health institution frequently would have higher chance of getting more comprehensive information from health professionals in the form of health education or counseling about pre-cervical cancer screening.

Getting information about cervical cancer from health providers was a significant predictor for uptake of pre-cervical cancer screening. Those participants who were informed by health providers about cervical cancer were 6.65 times higher to be screened as compared to their counter parts (AOR = 6.65, 95% CI = 3.64–12.15). This is in line with the studies done in Thailand, Portland Jamaica, Trelawney Jamaica, Uganda and Ethiopia [14, 17, 18, 26, 27]. This might be due to the fact that these women were more responsive to health providers who educate them about the disease and preventive measures. In addition, these discussions could be excellent opportunity to address negative attitudes about cervical cancer. Other explanation might be that women who were informed by health providers about cervical cancer could have high awareness and knowledge and they might be screened PCCS.

### Limitations of the study

The limitations of this study include that information was self-reported by participants and there was no way of verifying the screening.

### Recommendations

Federal Ministry of Health and Amhara Regional State Health Bureau should utilize mass media to influence the attitude of women towards cervical cancer and increase health-seeking behavior through health information dissemination. They should also encourage women to have annual health checkups as well as change a program that helps to include all reproductive age groups as target groups.

Zonal and Woreda Health Offices should strengthen information distribution about pre-cervical cancer screening by health providers throughout the community in places such as schools, markets and colleges to aid in changing the attitude of women to be receptive to pre-cervical cancer screening and promote youth-friendly service areas to appeal to young women.

Health institution and health professionals should strengthen the diagnosis and treatment of STIs for all women of reproductive age as a means to increase pre-cervical cancer screening indirectly and give PCCS information to all reproductive women that come for any purpose to any facilities.

Further studies should be conducted among reproductive women in rural areas about pre-cervical cancer screening, including a qualitative study to explore why there is a limited screening utilization.

## Conclusions

Pre-cervical cancer screening uptake was found to be low: Age group 35–49, informed by health professionals about cervical cancer, positive attitude to pre-cervical cancer screening, visited health institution, history of STI and family history of cervical cancer were a significant predictor for pre-cervical cancer screening uptake.

## Data Availability

The data sets generated during the study are available from the corresponding author upon request.

## Abbreviations

AOR: Adjusted Odd Ratio
CC: Cervical Cancer
COR: Crude Odd Ratio
ETB: Ethiopian Birr
HF: Health Facility
HIV: Human Immunodeficiency Virus
HP: Health Provider
HPV: Human Papilloma Virus
OCP: Oral Contraceptives Pills
PCCL: Precancerous Cervical Lesion
PCCS: Pre- Cervical Cancer Screening
SD: Standard Deviation
SPSS: Statistical Package for Social Sciences
SSA: Sub Saharan Africa
STI: Sexually Transmitted Infections
TV: Television
VIA: Visual Inspection with Acetic acid
WHO: World Health Organization
